# A general strategy to optimize immunogenicity of HLA-B*0702 restricted cryptic peptides from tumor associated antigens: Design of universal neo-antigen like tumor vaccines for HLA-B*0702 positive patients

**DOI:** 10.18632/oncotarget.11086

**Published:** 2016-08-05

**Authors:** Catherine Gallou, Aude Rougeot, Stéphanie Graff-Dubois, Kostas Kosmatopoulos, Jeanne Menez-Jamet

**Affiliations:** ^1^ Vaxon Biotech, 75015, Paris, France; ^2^ University Pierre-and-Marie-Curie (aka University Paris 06), INSERM U1135, CNRS ERL8255, 75013, Paris, France

**Keywords:** HLA-B7, vaccination, immunotherapy, optimized, cryptic

## Abstract

Tumor Associated Antigens (TAAs) are the privileged targets of almost all the cancer vaccines tested to date. Unfortunately all these vaccines failed to show a clinical efficacy. The main reason for this failure is the immune tolerance to TAAs that are self-proteins expressed by normal and cancer cells. Self-tolerance to TAAs is directed against their dominant rather than against their cryptic epitopes. The best way to overcome self-tolerance to TAAs would therefore be to target their cryptic epitopes. However, because of their low HLA-I affinity, cryptic peptides are non-immunogenic and cannot be used to stimulate an antitumor immune response unless their immunogenicity has been previously enhanced. In this paper we describe a general approach to enhance immunogenicity of almost all the HLA-B*0702 restricted cryptic peptides derived from TAAs. It consists in substituting residues at position 1 or 9 of low HLA-B*0702 affinity cryptic peptides by an Alanine or a Leucine respectively. These substitutions increase affinity of peptides for HLA-B*0702. These optimized cryptic peptides are strongly immunogenic and very importantly CTL they stimulate recognize their native counterparts.

TAAs derived optimized cryptic peptides can be considered as universal antitumor vaccine since they escape self-tolerance, are immunogenic and are not patient specific.

## INTRODUCTION

Immunotherapy based on Tumor Associated Antigens (TAAs) vaccination was a therapeutic approach which is the subject of a great interest during the last decade in the context of cancer treatment. The unique approach was to immunize with full length TAAs or derived antigenic peptides that stimulate Cytotoxic T Lymphocytes (CTLs), which play a major role in the elimination of tumor cells. Unfortunately, all preclinical and clinical studies were found to elicit only weak immunological responses with poor benefits in clinical studies [1–9]. Recently the use of MAGE full length antigen failed to reach the phase III objectives in the MAGRIT trial, the most important clinical study launched in cancer vaccination [9, 10].

Several factors may explain these disappointing results but the most likely is that dominant peptides targeted by all these vaccines are submitted to the central and peripheral self-tolerance, since all TAAs are non-mutated self-proteins expressed by normal tissues, including thymus. To overcome this issue, two ways were explored, i) recruiting the low affinity CTLs that could have escaped to central tolerance; major efforts were focused on the development of strong adjuvants that could strengthen immune response (for review see [11]); however the failure of the MAGE-A/ASCI vaccine that used a very powerful adjuvant showed that this way was wrong [9, 10], ii) identifying tumour specific immunogenic antigens. These antigens are abnormal proteins harbouring somatic mutations and are very often patient specific. All these mutations create T-cell reactive epitopes, called neo-antigens, which are recognized by the immune system as non-self and escape the self-tolerance process. Recently, the area of serial tumour genomic sequencing has allowed the identification of such neo-antigens and opened the way to personalized peptide based therapy [12]. However, neo-antigen must be identified for each patient individually that makes their use extremely difficult and expensive. Moreover, the use of neo-antigens as cancer vaccines may be limited by the genetic heterogeneity of tumors, indeed neo-antigens may be expressed by some but not all lesions of the same tumor.

We have previously described a new class of cancer vaccines. Like neo-antigens, these peptidic vaccines escape self -tolerance and are strongly immunogenic while, unlike neo-antigens, they can be used for the vaccination of all cancer patients.

These neo-antigen like universal cancer vaccines are called “optimized cryptic peptides”. Optimized cryptic peptides escape self-tolerance because they target TAAs derived cryptic peptides that are not involved in self-tolerance process and they are strongly immunogenic because their immunogenicity is optimized. The strategy to optimize immunogenicity of cryptic peptides, that because of their low MHC I affinity are non-immunogenic, consists in increasing their affinity for the MHC I molecules via amino acids substitutions [13]. These substitutions should however preserve the antigenicity of such optimized peptides. CTL generated by optimized peptides should indeed cross-react with the corresponding native peptides, which are presented at the tumor cell surface.

We have previously identified rules of selection and optimisation of HLA-A*0201 restricted cryptic peptides [8, 13], expressed by 45% of human population. Vx-001, the first optimized cryptic peptide based vaccine was already tested in a phase I/II study and was shown to be safe and strongly immunogenic in a majority of vaccinated patients [14–16]. Vx-001 is currently in Phase IIb double blind clinical study in stage IV NSCLC patients [17].

In this study, we investigated how to optimize tumor cryptic peptides presented by the HLA-B*0702 molecule, a CMH allele expressed by 25% of humans. We describe a general strategy to optimize HLA-B*0702 restricted cryptic peptides having a proline and an arginine at positions 2 and 3 respectively. It consists in substituting residues at positions 1 or C-terminal by an alanine and a leucine respectively.

This results open the way to the development of therapeutic vaccine designated to HLA-B*0702 cancer patients.

## RESULTS

### Selection of low HLA-B*0702 affinity non immunogenic peptides

It is well established that the immunogenicity of a peptide depends on its affinity for HLA molecules. High affinity peptides are immunogenic while low affinity peptides are not. HLA affinity depends on the presence/absence of primary anchor motifs and often on the presence of secondary anchor motifs that are both HLA allele specific. For instance peptides bound to HLA-B*0702 have a proline (P) and a leucine (L) or a methionine (M) at positions 2 and C-terminal respectively as primary anchor motifs. Regarding the secondary anchor positions, an alanine (A) at position 1 and an arginine (R) at position 3 are described to be favourable for binding to HLA-B*0702 [18].

We have previously described that the best method for identifying cryptic epitopes, that have a low HLA affinity consists in selecting from an antigen, peptides of 9/10 amino acids, having favourable primary anchor residues and non-favourable amino acids in at least one secondary anchor position [8, 13]. In the case of HLA-B*0702, optimally selected peptides must have a proline in position 2, and a leucine or a methionine in C-terminal position, and non-favourable amino acids at the secondary anchor position (1 and/or 3).

According to this rule, eight peptides with the HLA-B*0702 specific anchor motifs (P at position 2 and preferentially L/M at C-terminal position), derived from the universal antigens, Hsp70, TERT and the MAGE-A family (MAGE-A1, A2, A3, A4, A6, A10 and A12) were designed *in silico* and tested for binding to the HLA-B*0702 molecule. One peptide (TERT_4_) harbouring the complete consensus motif *i.e.* A1P2R3L9 was also selected as a control.

Selected cryptic peptides were very weak or even non HLA-B*0702 binders (Table 1), with relative affinity not evaluable (superior to 10). This confirms the importance of the secondary anchor residues since the presence of primary anchor motifs is not sufficient by itself to ensure a high binding affinity to HLA-B*0702. TERT_4_ that harbours the consensus sequence was shown to strongly bind to HLA-B*0702 (Table 1).

**Table 1:** HLA-B*0702 affinity of peptides

antigen	peptide	séquence	RA
TERT	TERT_4_	APRCRAVRSL	0.74 ± 0.12
Hsp70	Hsp70_115_	YPEEISSMVL	≥ 10
	Hsp70_137_	YPVTNAVITV	≥ 10
	Hsp70_397_	APLSLGLET	≥ 10
TERT	TERT_444_	DPRRLVQLL	≥ 10
MAGE-A	MAGE-A1_121_	EPVTKAEML	≥ 10
	MAGE-A2_121_	EPFTKAEML	≥ 10
	MAGE-A4_121_	EPITKAEIL	≥ 10

RA: Relative Affinity; ≥ 10 means that RA can be 10 or not measurable because the affinity is less than 20% of the standard peptide affinity. Each experiment was reproduced at least three times giving similar results, mean values and standard deviation are reported in the table.

Given their low affinity, peptides Hsp70_115_, Hsp70_137_, Hsp70_397_, TERT_444_, MAGE-A1_121_, MAGE-A2_121_ and MAGE-A4_121_ are considered cryptic peptides.

TERT_4_ in contrast has a strong affinity for HLA-B*0702, and is considered dominant peptide.

**Figure 1:**
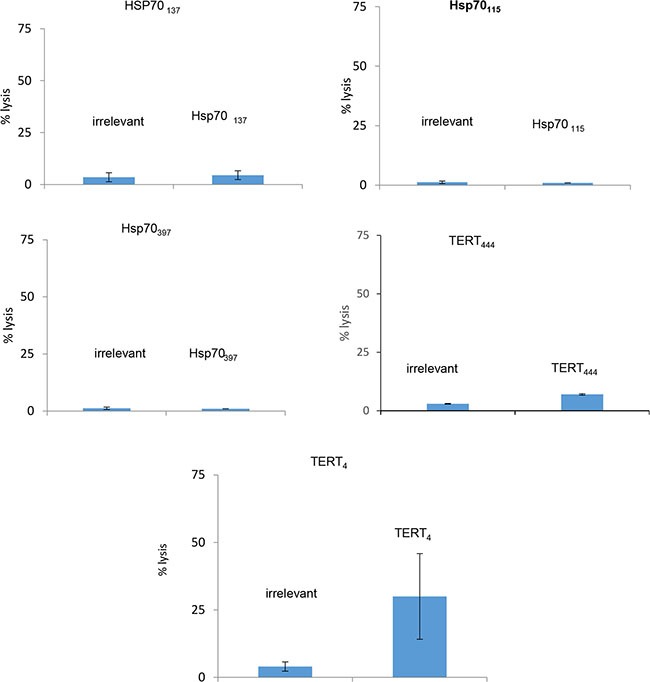
Cytotoxicity of murine CTLs from HLA-B*0702 transgenic mice vaccinated with native peptides Six HLA-B*0702 transgenic mice were vaccinated with each of the selected native peptides. Specific CTLs induction was evaluated by the capacity of lymphocytes from spleen cells to lyse target cells loaded with an irrelevant peptide or the peptide used for vaccination (ratio 30/1).

The low affinity Hsp_137_, Hsp_115_, Hsp_397_, TERT_444_ and the high affinity TERT_4_ peptides have been tested for their capacity to induce a specific CTL immune response in humanized HLA-B*0702 transgenic mice. As expected only the high affinity TERT_4_ was immunogenic confirming that immunogenicity of peptides is strongly related to their affinity for HLA (Figure 1).

### Optimization of HLA-B*0702 affinity and *in vivo* immunogenicity of peptides harbouring substitutions at secondary anchor positions

Since all these cryptic peptides had favourable primary anchor motifs, enhancement of their affinity that is a prerequisite for them to be immunogenic, would require the modification of unfavourable secondary anchor motifs and their substitution with favourable amino acids. These substitutions should however preserve the conformation of the peptide segment that interacts with the TCR (usually position 3 to position 8 for a 9-mer).

As previously described, an alanine at position 1 and an arginine at position 3 are favourable [18]. Our interest was, therefore, focused on secondary anchor positions 1 and 3.

For cryptic peptides Hsp_115_, Hsp_397_, TERT_444_ and MAGE-A1_121_ non-favourable secondary anchor motifs were respectively replaced by an alanine at position 1 and arginine at position 3 (Table 2). For Hsp70_397_ the unfavourable leucine at position 3 was replaced by an arginine and an additional modification (replacement of a threonine by a leucine) was brought at the C-terminal position to increase the predicted immunogenicity.

**Table 2:** HLA-B*0702 affinity of peptides

Peptide	sequence	RA
Hsp70_115_	YPEEISSMVL	≥ 10
Hsp70_115_ A1	Apeeissmvl	≥ 10
Hsp70_115_ A1R3	ApReissmvl	1.6 ± 0.97
Hsp70_397_	APLSLGLET	≥ 10
Hsp70_397_ R3L9	APRSLGLEL	1 ± 0.6
TERT_444_	DPRRLVQLL	≥ 10
TERT_444_A1	APRRLVQLL	1.4 ± 0.83
MAGE-A1_121_	EPVTKAEML	≥ 10
MAGE-A1_121_ A1	APVTKAEML	≥ 10
MAGE-A1_121_ A1R3	APRTKAEML	2.36 ± 0.28

RA: Relative affinity of cryptic and optimized peptides; ≥ 10 means that RA can be 10 to not measurable because it is not possible to determine the 20% of standard value. Each experiments was reproduced at least three times giving comparable results, mean values and standard deviation are reported in the Table.

Results presented in Table 2 show that the presence of a favourable motif at secondary positions 3 is absolutely necessary for a peptide to have a high HLA-B*0702 affinity. This is the case of peptides Hsp70_115_A1R3, Hsp70_397_R3L9, TERT_444_A1, MAGE-A1_121_A1R3. It is noteworthy that the high affinity TERT_4_ has favourable residues at both positions 1 and 3. Inversely peptides with favourable residue at only position 1 fail to bind to HLA-B*0702 with high affinity. This is the case of Hsp70_115_A1 and MAGE-A1_121_A1.

These results suggest that binding motifs for HLA-B*0702 are more stringent than for HLA-A*0201 for which position 3 is not determinant and suggest that for HLA-B*0702 position 3 could be considered as a primary anchor position.

Optimized cryptic peptides Hsp70_115_A1R3, Hsp70_397_R3L9 and TERT_444_A1 were then tested in HLA-B*0702 transgenic mice for their capacity to stimulate CTL able to recognize their native counterpart.

**Figure 2:**
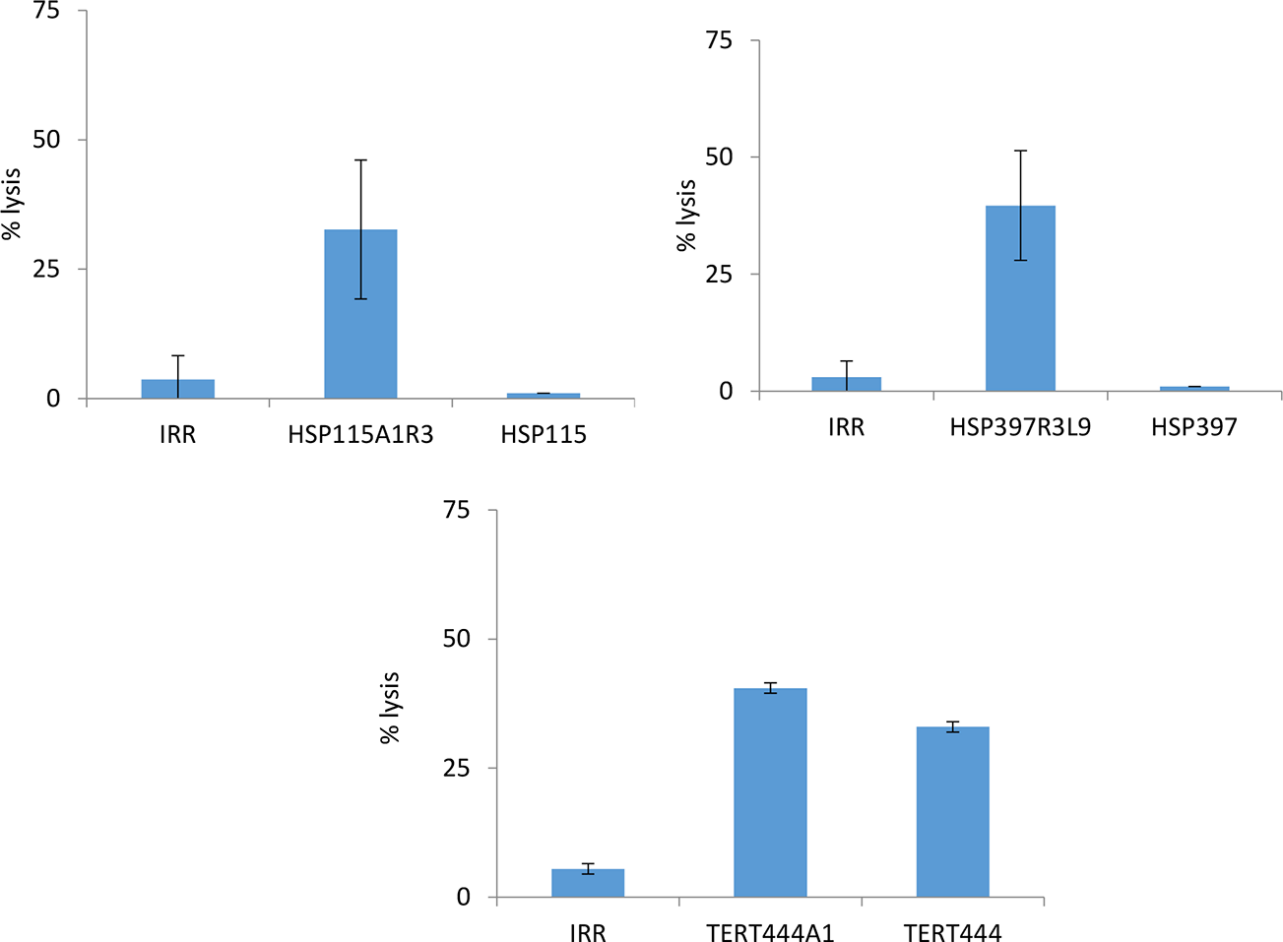
Cytotoxicity of murine CTLs from HLA-B*0702 transgenic mice vaccinated with optimized peptides Three to six HLA-B*0702 transgenic mice were vaccinated with optimized peptides, and specific CTLs were tested against target cells loaded with an irrelevant peptide, the optimized peptide used for vaccination or the corresponding native peptide (ratio 30/1).

Figure 2 shows that all tested high affinity optimized peptides were strongly immunogenic confirming once again the correlation between high binding affinity and immunogenicity.

However, for all these peptides but TERT_444_A1, generated CTL recognized target cells loaded with the optimized peptide but not the corresponding native peptide. This strongly suggests that substitution of residues at position 3 changes the conformation of the peptide segment that interacts with the TCR and guarantees the TCR cross-recognition of the native counterpart.

Since the native peptide cross-recognition by the optimized peptide induced CTL is mandatory for a vaccine candidate, because the native peptide is the only one that is expressed at the tumour cell surface, new rules of native peptide selection and optimization were investigated.

### Selection of cryptic peptides with favourable amino acids at positions 2 and 3

Since a) the presence of favourable residues at both position 1 and 3 is necessary for a peptide to strongly bind to HLA-B*0702 and b) a modification of the residue at position 3 alters the interaction of the specific TCR and the HLA-B*0702/peptide complex thus preventing the recognition of the native counterpart, we proceeded to the selection of peptides that have favourable residues at position 2 and 3 and unfavourable residues at position 1 or 9. Theoretically, these peptides should have a low affinity for HLA-B*0702 and optimization of their affinity should require modification of the residue at position 1 and/or 9 that should not have an impact on the TCR/HLA-B*0702/peptide interaction. Except a proline, no other aminoacids are described to be unfavourable at position 1 (http://www-bimas.cit.nih.gov/cgi-bin/molbio/hla_coefficient_viewing_page). In this context, the probability to select high affinity immunogenic native peptide increases dramatically.

Ten peptides were selected according to these rules, HER2/neu_246_, HER2/neu_760_, HER2/neu_1069_, HER2/neu_1151_, HSP70_466_, CEA_188,_ P53_63_, MAGE-A1_267_, MAGE-A2_274_ and MAGE-A3_274_ and directly tested in HLA-B*0702 transgenic mice to rapidly select non immunogenic peptides (Table 3). It is noteworthy that each peptide sequence derived from MAGE-A is present in two genes of the MAGE-A family: MAGE-A1_267_ is shared by MAGE-A1 and MAGE-A4, MAGE-A2_274_ is shared by MAGE-A2 and MAGE-A6 and MAGE-A3_274_ is shared by MAGE-A3 and MAGE-A12. Moreover, all these sequences are homolog and differ only at position 6 (Table 3).

**Table 3:** Immunogenicity of native HLA-B*0702 restricted peptides, number of responding mice/number of vaccinated mice, peptides in grey were withdrawn from the selection process, *MAGE-A3_274_ was selected for further optimization because the immune response it generated was very weak (see Figure 3)

Antigen	Peptide	Sequence	Immunogenicity
Her2/neu	Her2/neu_760_	SPKANKEIL	4/9
	Her2/neu_246_	GPKHSDCLA	0/9
	Her2/neu_1069_	APRSPLAPS	5/7
	Her2/neu_1151_	SPREGPLPA	1/3
MAGE-A1; MAGE-A4	MAGE-A1_267_	GPRALAETS	0/3
MAGE-A2; MAGE-A6	MAGE-A2_274_	GPRALIETS	0/3
MAGE-A3, MAGE-A12	MAGE-A3_274_	GPRALVETS	2/3*
CEA	CEA_188_	SPRLQLSND	0/3
HSP70	HSP70_466_	APRGVPQIEV	3/3
P53	P53_63_	APRMPEAAP	3/3

**Figure 3:**
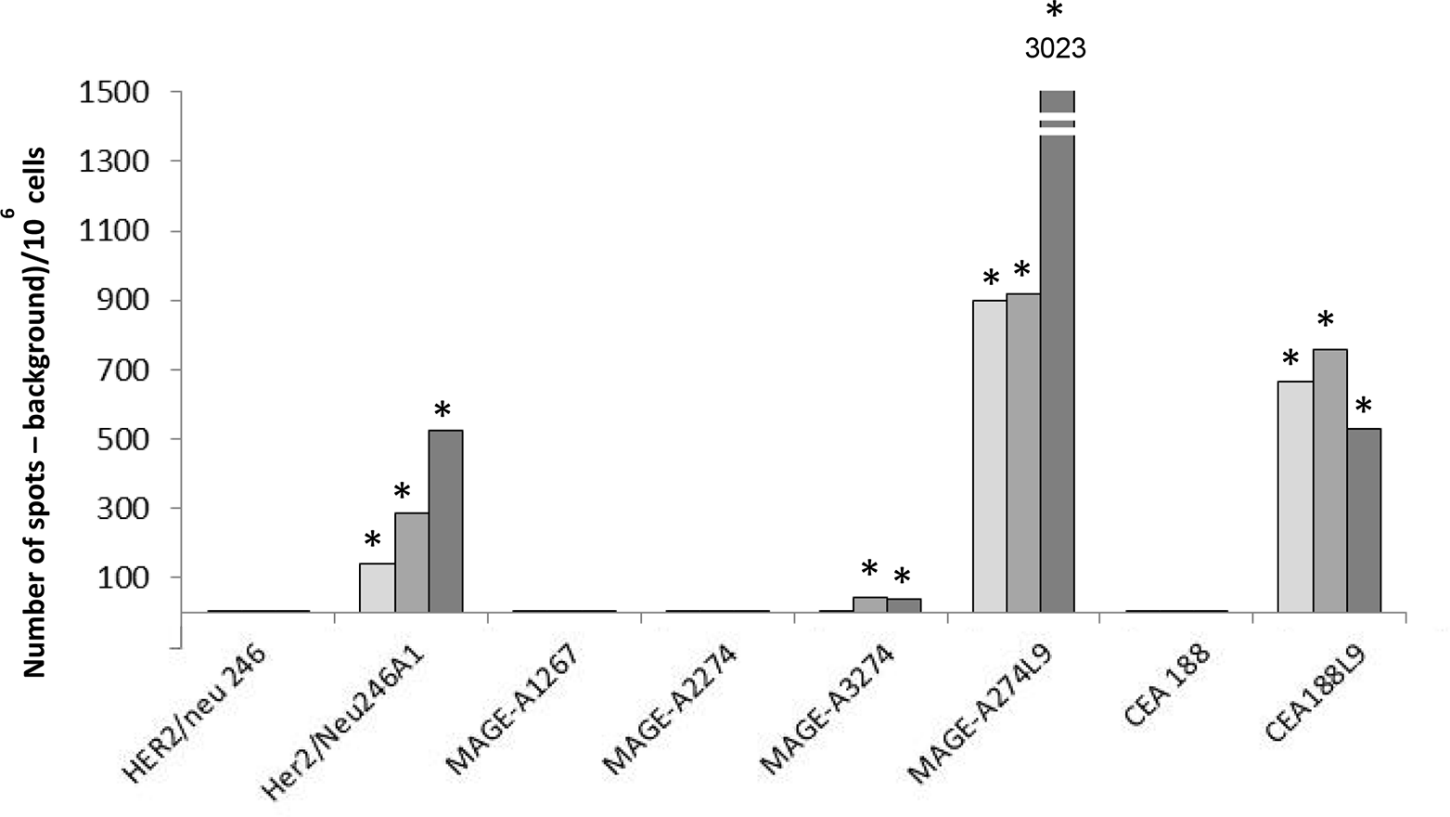
Immunogenicity of selected native and optimized peptides in HLA-B*0702 transgenic mice Three HLA-B*0702 transgenic mice (clear, medium and dark gray respectively represents results of one mouse) were vaccinated twice at two weeks interval with the native or the optimized peptide. For each mouse, the number of specific CTLs was determined by ELISpot assay and expressed per 10^6^ cells. * means that difference are statistically significant from negative control (*p* ≤ 0.05).

HER2/neu_760,_ HER2/neu_1151_, HSP70_466_, P53_63_ and HER2/neu_1069_ native peptides were shown to be immunogenic in HLA-B*0702 transgenic mice and were withdrawn from the selection process. In contrast, HER2/neu_246_, MAGE-A1_267_, MAGE-A2_274_ and CEA_188_ were non immunogenic and were selected to be further optimized (Table 3 and Figure 3).

A very weak immunogenicity (see Figure 3) was observed with MAGE-A3_274_ which, in spite of that, was selected for further optimisation.

### Optimization of HLA-B*0702 affinity and *in vivo* immunogenicity of non-immunogenic cryptic peptides with favourable amino acids at positions 2 and 3

Optimized peptides Her2/neu_246_A1 modified at position 1 by an alanine and CEA_188_L9, modified at last position by a leucine were tested in HLA-B*0702 transgenic mice.

MAGE-A_274_L9 was derived from MAGE-A3_274_, since MAGE-A3_274_ is shared by MAGE-A3, the most expressed in tumour cells (46% in lung, 57% in gastric cancer, 68% in hepatocarcinoma, 76% in RCC) and MAGE-A12 expressed in 74% of melanoma. MAGE-A_274_L9 optimized peptide was tested in HLA-B*0702 transgenic mice for immunogenicity and cross recognition of the corresponding native MAGE-A3_274_ peptide and also against the two homolog native sequences MAGE-A1_267_ and MAGE-A2_274_.

Figure 3 shows that HER2/neu_246_A1, CEA_188_L9 and MAGE-A_274_L9 optimized peptides are immunogenic in HLA-B*0702 transgenic mice. More importantly, induced CTLs are able to recognize target cells loaded with their corresponding native peptide. MAGE-A_274_L9 induced CTLs were shown able to recognize target cells loaded with MAGE-A3_274_ but also MAGE-A1_267_ and MAGE-A2_274_ homolog sequences (Table 4).

**Table 4:** Immunogenicity of optimized HLA-B*0702 restricted peptides and cross recognition of the native peptides

Antigen	Peptide	Sequence	Immunogenicity against optimized peptide	Immunogenicity against native peptide
HER2/neu	HER2/neu_246_A1	APKHSDCLA	8/12	5/12
MAGE-A	MAGE-A_274_ L9	GPRALVETL	18/18	MAGE-A1_267_ 3/8 MAGE-A2_274_ 3/5 MAGE-A3_274_ 4/5
CEA	CEA_188_L9	SPRLQLSNL	6/6	6/6

HLA-B*0702 mice were vaccinated twice with the optimized peptides. Specific CTLs were detected by cytotoxicity or IFNγ ELISPOTs against target cells loaded with the optimized peptide or the native peptide, results are presented as number of responding mice/number of vaccinated mice.

### Optimized cryptic peptides are immunogenic *in vitro* in human

Four couple of native/optimized peptides previously validated in HLA-B*0702 transgenic mice: TERT_444_-TERT_444_A1, HER2/neu_246_-HER2/neu_246_A1, MAGE-A3_274_/MAGE-A1_267_/MAGE-A2_274_–MAGE-A_274_L9 and CEA_188_-CEA_188_L9 were tested *in vitro* for their capacity to induce specific CTLs from healthy PBMC donors.

CD8+ T cells from healthy donors were *in vitro* stimulated with autologous dendritic cells loaded with each optimized cryptic peptide. After four stimulations, proliferating cells were tested for the presence of IFNγ producing CTLs upon stimulation with T2-B7 cells loaded with optimized or native peptides either by intracellular IFNγ staining or by IFNγ ELISPOT assay.

Table 5 summarizes experiments with TERT_444_A1, HER2/neu_246_A1, MAGE-A_274_L9 and CEA_188_L9. Immunogenicity of TERT_444_A1, HER2/neu_246_A1, MAGE-A_274_L9 and CEA_188_L9 was confirmed. More importantly, the specific CTLs induced by vaccinating with the optimized peptides were able to recognize target cells loaded with the optimized or with the corresponding native peptide confirming that optimized peptide specific CTLs are able to recognize the corresponding native peptide.

**Table 5:** Immunogenicity of optimized HLA-B*0702 restricted peptides, and cross recognition of the native peptide by CTLs induced by the optimized peptide in PBMC from human healthy donors; number of positive culture/number of total cultures

Peptide	Sequence	Immunogenicity against optimized peptide	Immunogenicity against native peptide
TERT_444_A1	APRRLVQLL	1/2	2/2
HER2/neu_246_A1	APKHSDCLA	2/4	1/4
MAGE-A_274_ L9	GPRALVETL	2/4	MAGE-A1_269_ 0/4 MAGE-A2_274_ 2/4 MAGE-A3_274_ 2/4
CEA_188_L9	SPRLQLSNL	1/3	2/3

**Figure 4:**
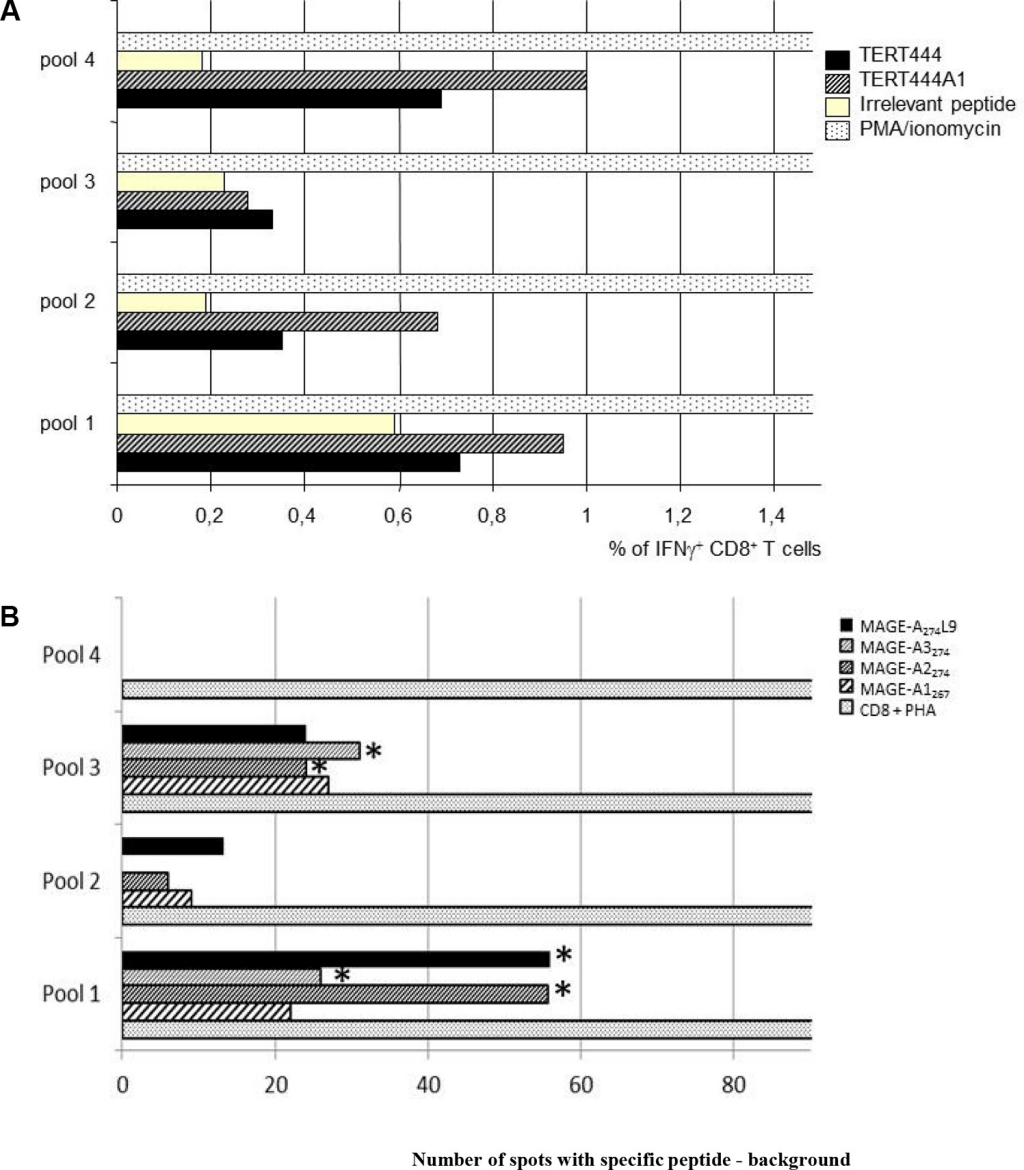
Immunogenicity of selected optimized cryptic peptides in human PBMC culture PBMC from HLA-B*0702 healthy volunteers were stimulated 5 weeks with optimized cryptic peptides TERT_444_A1 or MAGE-A_274_L9 in presence of monocyte-derived dendritic cells. CD8^+^ were divided in 4 pools (numbered pool 1 to pool 4) and tested for the recognition of the optimized or the corresponding native peptides. (**A**) % of CD8^+^ IFNg producing CTLs after stimulation with TERT_444_A1 determined by INFg intracellular staining. PBMCs were divided in 4 pools (numbered pool 1 to pool 4) and tested for the production of intracellular IFNg in presence of TERT_444_A1 or TERT_444_ native peptide. This experiment was reproduced twice with PBMCs from two different donors giving similar results. (**B**) ELISpot assay of CD8^+^ T cells derived from human PBMC stimulated with MAGE-A_274_L9. PBMCs were divided in 4 pools (numbered pool 1 to pool 4) and tested for the recognition of MAGE-A_274_L9 or for MAGE-A_274_ native variants. ELISpot assays were performed in sixplicate wells and a t test was done to evaluate the difference between the mean of number of spots in the negative control (cells loaded with an irrelevant peptide) and the mean of number of spots with the specific peptides. * means that difference are statistically significative (*p* ≤ 0.05). This experiment was reproduced four times with PBMCs from four different donors.

Regarding MAGE_273_L9, the cross recognition of MAGE-A3_274_ and MAGE-A2_274_ was confirmed but not for MAGE-A1_267_ (Figure 4).

Figure 4 presents two representative experiments. Figure 4A shows that IFNγ producing CTLs were detected by IFNγ intracellular staining in pools 2 and 4 after stimulation with either TERT_444_A1 or TERT_444_ loaded T2B7 cells confirming the cross recognition of the native peptide by CTLs stimulated with the optimized one. Figure 4B shows a representative experiment with MAGE-A_274_L9. Significant specific immune responses were observed against MAGE-A2_274_ and MAGE-A3_274_ in pool 1 and 3 confirming the native peptides recognition. Cumulative experiments are presented in Table 5. As mentioned before MAGE-A1_267_ was not recognized significantly in these experiments even if specific CTLs were detected (Figure 4B).

## DISCUSSION

Despite a very promising concept, cancer vaccination gave disappointing clinical results in cancer patients. All cancer vaccines tested to date targeted TAAs that are self-proteins expressed by both tumor and normal cells. The main reason that explains the failure of cancer vaccines is immune tolerance to the self-antigens they targeted (self-tolerance). The necessity to circumvent tolerance to self tumor antigen was recently taken into account with a new class of cancer antigens: the neo-antigens. Neo-antigens are mutation harbouring tumor epitopes, which are recognized by the immune system as non-self. Indeed, their specific T cell repertoire is not tolerized and is therefore available for specific stimulation.

Neo-antigens could then be very promising candidate in tumor vaccination providing that they are strongly immunogenic and showed really convincing results in preclinical models [19] and recently in humans in early stage but promising clinical studies [20–22].

However, the use of neo-antigens in cancer vaccination can be limited by two factors. First, the genetic heterogeneity of tumors; it is likely that neo-antigens are not expressed by all lesions of the same tumor. Neo-antigen free lesions will not be targeted by neo-antigen specific T cells. Second, neo-antigens are patient specific and their use requires their previous identification in each patient individually. This can be done only in highly specialized research centers and is time consuming and expensive. Moreover, since neo-antigens are patient specific they will be administered to the individual patient without any previous demonstration of safety and efficacy in preclinical and clinical studies.

It seems therefore mandatory to find tumor antigens that have the two main properties of neo-antigens (escape self-tolerance and strong immunogenicity) while they are not patient specific and can therefore be used for treatment of all cancer patients. In several previous paper we showed that optimized TAAs derived cryptic peptides escape self-tolerance, are immunogenic and can be used for the treatment of all cancer patients [8, 13]. They can therefore be considered as universal neo-antigen like peptide vaccines. Importantly, optimized cryptic peptides based vaccines can undergo a classical preclinical and clinical development to evaluate safety and efficacy before becoming a cancer treatment.

Tumor cryptic peptides although they escape self-tolerance are not immunogenic because of their low HLA-I affinity. Their immunogenicity should be optimized before their use in cancer vaccination. We have previously shown that a substitution of the first amino acid, whatever is this amino acid by a tyrosine optimizes immunogenicity of almost all HLA-A*0201 restricted cryptic peptide [13]. Such optimized cryptic peptides have been successfully used in preclinical models and in cancer patients. The first anticancer therapeutic vaccine based on optimized cryptic peptide, Vx-001 was tested in a phase I/II clinical study in 116 cancer patients showing no toxicity and frequent specific immune response [14–16]. Vx-001 is currently in a randomized phase IIb in metastatic or recurrent NSCLC patients [17]. Vx-001 is designated to HLA-A*0201 expressing patients representing 45% of humans. Developing optimized cryptic peptide based vaccine for patients expressing other HLA molecules is then necessary to increase the proportion of patients suitable to be treated with such products.

The strategy for enhancing the immunogenicity of cryptic peptides, that because of their low MHC I affinity are non-immunogenic, consists in increasing their affinity for the MHC I molecules through amino acids substitutions [13] while preserving the antigenicity of such optimized peptides. CTL generated by optimized peptides should indeed cross-react with the corresponding native peptides, which are presented at the tumor cell surface. Peptide affinity for MHC I molecules mainly depends on the presence at well-defined positions of residues called “primary anchor residues”, which are MHC I allele specific. Other residues at positions called “secondary anchor positions” that are less HLA specific may play a role in the HLA/peptide interaction. The rules to select and optimized cryptic peptides is then totally different for each HLA molecule. In this study, we have defined how to design optimized cryptic peptides for HLA-B*0702, allele that is expressed by 25% of human population.

High affinity HLA-B*0702/peptide interaction requires the presence of a proline and a leucine at primary anchor positions 2 and c-terminal respectively as well as an alanine and a lysine/arginine at secondary positions 1 and 3 respectively. Our initial strategy was to select HLA-B*0702 restricted cryptic peptides having at least one primary anchor motif (Proline at position 2 and if possible leucine at position 9) and unfavourable residues at position 1 and position 3. Thus substituting unfavourable residues at the two secondary positions with favourable ones should enhance HLA-B*0702 affinity and immunogenicity of selected cryptic peptides. As expected all selected peptides exhibited a low affinity which is the main feature of a cryptic peptide. However, substitutions of residues at position 3 although they enhanced affinity of all tested peptides led to the loss of the cross-recognition of the native cryptic peptide. This is probably due to the fact that position 3 is close to the peptide segment that interacts with the T Cell Receptor (position 4 to position 8) and substitution at position 3 alters the conformation of this TCR contacting peptide segment. Since the absence of a favourable residue at position 3 (lysine or arginine) seems to be deleterious for the HLA-B*0702/peptide interaction (compare affinity of Hsp70_115_A1-Hsp70_115_A1R3 and MAGE-A1_121_A1 - MAGE-A1_121_A1R3) we decided to select cryptic peptide candidates based on the presence of a proline at position 2, a Lysine/Arginine at position 3 and an unfavorable residue at position 1. Almost all peptides having this profile were non-immunogenic (TERT_444_, HER-2/neu_246_, MAGE-A1_267_, MAGE-A2_274_, CEA_188_) or were very weakly immunogenic (HER-2/neu_1151_ and MAGE-A3_274_). Importantly immunogenicity was restored when an alanine has substituted the residue at position 1 demonstrating the importance of this position in the HLA-B*0702/peptide interaction. CTLs induced by all these optimised peptides recognized their native counterpart. This is easily explained by the fact that position 1 is separated from the TCR contacting segment of the peptide by two anchor positions 2 and 3 that protect this segment from any modification brought at position 1.

Immunotherapy is now considered as a really promising approach to treat cancer. Recently, the discovery and development in this domain was largely focused on immune checkpoint inhibitors such as antiCTLA-4 or anti PD-1/PD-L1 [23].

PD-1 is expressed on activated T cells. It is known to play a major role in tumor escape since after binding to its ligands PD-L1 or PD-L2, PD-1 down regulates signalling by the TCR leading to immune suppression by T cell anergy or apoptosis [24].

Inhibition of the PD-1/PD-L1 pathway leads to the suppression the tumor microenvironment negative effect on the immune response. However, PD-1 inhibitors are very weakly or not at all efficient in the absence of tumor infiltrating T cells that are frequent in tumors with high mutational load.

In this context inducing a strong immune response with optimized cryptic peptides that targets antigens expressed by all tumor cells, could potentiate the action of immune checkpoint inhibitors by inducing tumor specific T cells that could infiltrate the tumor. Moreover, optimized cryptic peptide induced immune response will render sensitive to PD-1 inhibitors treatment tumors that are naturally non immunogenic because they have low mutational load and are not therefore infiltrated by T Infiltrating Lymphocytes (TILs). Thus, *c*ombining optimized cryptic peptide based vaccine and immune checkpoint inhibitors could then increase dramatically the proportion of patients that could benefit from these treatments.

In this study, we describe how to optimize immunogenicity of HLA-B*0702 restricted cryptic peptides from TAAs.

These results open the way for the development of universal neo-antigen like therapeutic vaccine designated to HLA-B*0702 patients, representing at least 25% of the human population, which have the two main properties of neo-antigens -they escape self-tolerance and are immunogenic- while unlike neo-antigens they are not patient specific and can be used for vaccination of all cancer patients.

## MATERIALS AND METHODS

### Transgenic mice

The HLA-B7 H-2 class-I knockout mice were previously described [25].

### Cells

HLA-B*0702 transfected murine RMA-B7 and human T2-B7 cells were previously described [25].

### Peptides and plasmids

Peptides were synthesized by Millegen (Labège, France).

### Measurement of peptide relative affinity to HLA-B*0702

The protocol used has been described previously [25]. Briefly, T2-B7 cells were incubated at 37°C for 16 hours with peptides concentrations ranging from 100 μM to 0.1 μM (100 μM, 10 μM, 1 μM and 0.1μM), and then stained with ME-1 monoclonal antibody (mAb) to quantify the surface expression of HLA-B*0702. For each peptide concentration, the HLA-B*0702 specific staining was calculated as the percentage of staining obtained with 100 μM of the reference peptide CMV_265–274_ (R10V; RPHERNGFTV, SEQ ID NO: 9). The relative affinity (RA) was determined as: RA = (Concentration of each peptide that induces 20% of HLA-B*0702-expression/Concentration of the reference R10V peptide that induces 20% of HLA-B*0702 expression). In case of non-binders peptide, RA is not evaluable since the 20% of HLA-B*0702 expression is not reach even with the highest concentration of peptide.

### Vaccination of HLA-B*0702 transgenic mice

Mice were injected subcutaneously with 100 μg of peptide emulsified in Incomplete Freund's Adjuvant (IFA) in the presence of 150 μg of the I-A^b^ restricted HBVcore_128_ T helper epitope (TPPAYRPPNAPIL) twice at two weeks interval.

A particular protocol was applied for MAGE-A _274_L9, with two injections of this peptide at two weeks interval and 15 days later one injection with each one of the native peptide.

### Cytotoxic assay

Eleven days after the last vaccination 5 × 10^7^ spleen cells were *in vitro* stimulated with the corresponding native peptide (10 μM) in RPMI 1640 medium supplemented with 10% FCS, 2 μM glutamine and antibiotics. On day 6 of culture the bulk responder populations were tested for specific cytotoxicity. Target cells were labelled with 100 μCi of ^51^Cr for 60 min, plated in 96-well V-bottomed plates (3 × 10^3^ cell/well in 100 μL of RPMI 1640 medium) and, when necessary, pulsed with peptides of interest or an irrelevant peptide (1 μM, irrelevant peptide is not restricted by HLA-B*0702) at 37°C for 2 hours. Effectors were then added in the wells and incubated at 37°C for 4 hours at a ratio of 30 effector cells for 1 target cell. Percentage of specific lysis was determined as: % Lysis = (Experimental Release-Spontaneous Release) / (Maximal Release-Spontaneous Release) × 100. Spontaneous release is obtained using medium only, maximum release with HCl 0.1 M.

### Murine IFNγ ELISPOT assay

Seven days after the last vaccination, spleens were removed and T cells were isolated by Ficoll centrifugation and tested *ex vivo* for the recognition of 10 μM of either the native or optimized peptide. IFNγ producing T cells were quantified by ELISpot using the Diaclone Kit Murine IFNγ ELISpot (Besançon, France) according to manufacturer's recommendations. 250 000 spleen T cells were distributed per well and tested for the recognition of 10 μM of either the native or optimized peptide. Positive control was Concanavalin A and negative control was medium alone. All conditions were tested in sixplicates.

### Generation of CTL from human PBMC

PBMC were collected by leukapheresis from healthy HLA-A*B702 volunteers. Dendritic cells (DC) were produced from adherent cells cultured with 500 IU/ml GM-CSF and 500 IU/ml IL-4, in complete synthetic medium (AIMV) for seven days. On day 7, DCs were pulsed with 10 μM of peptide during 2 hours and CD8^+^ cells were purified by negative selection with CD8 Dynabeads Untouched Human CD8. CD8^+^ cells (2 × 10^5^) + CD8^−^ cells (6 × 10^4^) were stimulated with 3 × 10^4^ peptide-pulsed DC in AIMV medium supplemented with 1000 IU/ml IL-6 and 5 IU/ml IL-12 in round-bottomed 96-well plates.

From day 7, cultures were stimulated weekly with peptide-loaded DC in the presence of 20 IU/ml IL-2 and 10 ng/ml IL-7, 1 μg/ml anti-CD40 and 500 IU/ml IFNγ.

After the third stimulation, CD8 cells were maintained in AIMV supplemented with 20 IU/ml IL-2 during 7 days. Cultures were starved overnight with AIMV and then were tested in an intracellular IFNγ and an IFNγ ELISpot assay.

### IFNγ intracellular staining

Stimulated T cells (10^5^) from each pool were incubated with 2 × 10^5^ T2 cells loaded with stimulating peptide in the presence of 20 μg/ml Brefeldin-A (Sigma, Oakville, Canada). Six hours later they were washed, stained with r-phycoerythrin-conjugated anti-CD8 antibody (Caltag Laboratories, Burlingame, CA, USA) in PBS for 25 min at 4°C, washed again, and fixed with 4% PFA. The cells were then permeabilized with PBS, 0.5% BSA, 0.2% saponin (Sigma, Oakville, Canada), and labelled with an allophycocyanin-conjugated anti-IFNγ mAb (PharMingen, Mississauga, Canada) for 25 min at 4°C before analysis with a FACSCalibur^®^ flow cytometer.

### Human IFNγ ELISPOT assay

Stimulated T cells were splited in four pools and IFNγ producing T cells was quantified in each pool by ELISpot using the Diaclone Kit Human IFNγ ELISpot (Besançon, France) according to manufacturer's recommendations. 250 000 cells were distributed per well and tested for the recognition of 10 μM of either the native or optimized peptide. Positive control was Concanavalin A and negative control was medium alone or an irrelevant peptide. All conditions were tested in sixplicates.
